# Characterization of a Tumor-Associated Activating Mutation of the p110β PI 3-Kinase

**DOI:** 10.1371/journal.pone.0063833

**Published:** 2013-05-29

**Authors:** Hashem A. Dbouk, Bassem D. Khalil, Haiyan Wu, Aliaksei Shymanets, Bernd Nürnberg, Jonathan M. Backer

**Affiliations:** 1 Department of Molecular Pharmacology, Albert Einstein College of Medicine, Bronx, New York, United States of America; 2 Department of Pharmacology and Experimental Therapy, Institute for Pharmacology and Toxicology and Interfaculty Center of Pharmacogenomics and Pharma Research Eberhard-Karls-Universität Tübingen, Tübingen, Germany; University of Torino, Italy

## Abstract

The PI3-kinase pathway is commonly activated in tumors, most often by loss of PTEN lipid phosphatase activity or the amplification or mutation of p110α. Oncogenic mutants have commonly been found in p110α, but rarely in any of the other catalytic subunits of class I PI3-kinases. We here characterize a p110β helical domain mutation, E633K, first identified in a Her2-positive breast cancer. The mutation increases basal p110β activity, but does not affect activation of p85/p110β dimers by phosphopeptides or Gβγ. Expression of the mutant causes increases in Akt and S6K1 activation, transformation, chemotaxis, proliferation and survival in low serum. E633 is conserved among class I PI3 Ks, and its mutation in p110β is also activating. Interestingly, the E633K mutant occurs near a region that interacts with membranes in activated PI 3-kinases, and its mutation abrogates the requirement for an intact Ras-binding domain in p110β-mediated transformation. We propose that the E633K mutant activates p110β by enhancing its basal association with membranes. This study presents the first analysis of an activating oncogenic mutation of p110β.

## Introduction

The PI3-kinase signaling pathway is inappropriately activated in a variety of tumors [1]. Hyperactivation of the pathway is commonly caused by mutation or deletion of the Phosphatase and Tensin Homolog (PTEN), which dephosphorylates the PI3-Kinase product PIP3 to generate PIP2. Activating mutations of p110α [2], oncogenic mutations in the regulatory p85 subunits [3], as well as amplification of the catalytic subunits [4], [5], have also been documented. Significantly, mutations in the other class I catalytic subunits, p110β, p110δ or p110γ, are rarely seen in tumors. However, unlike p110α, which is only transforming when mutated, over-expression of the wild-type forms of p110-β, -δ or -γ cause transformation [6]. The ability of p110β to transform in the wild-type state has been attributed in part to decreased basal inhibition of p110β activity by p85 [7], although this has been controversial [8], [9]. In addition, a recent study has shown a requirement for Gβγ inputs to p110β for cellular transformation, particularly in PTEN-null tumors [10].

This study is the first characterization of a tumor-associated p110β mutation. The mutation, E633K, was identified in a HER2-positive breast tumor [11]. We show that this helical domain mutation increases basal activity of p110β and enhances its transforming potential *in vitro*. In addition, cells stably expressing this mutation display faster proliferation, enhanced survival in low serum, and increased motility. The region containing this mutation is an acidic patch that is in close proximity to the ABD-RBD linker and the RBD domain of p110β, and it is conserved in all class I PI3Ks. Our data suggests a novel inhibitory interface that can be disrupted in tumors.

## Materials and Methods

### p110 Constructs

myc-p110 constructs were mutated using Quickchange site-directed mutagenesis (Stratagene, CA).

### Cell Culture & Transfections

HEK293T cells were cultured in DMEM/10% FBS. NIH 3T3 cells were cultured in DMEM/10% NCS. Cells were transfected with equal amounts of p85 or p110-myc using Fugene HD (Promega) according to manufacturer’s instructions. For generation of stably-expressing cell lines, NIH 3T3 were transfected with wild type or E633K C-myc p110β in pcDNA3.1 and then selected using G418 (800 µg/ml), and maintained in 200 µg/ml of G418.

### Expression and Purification of Recombinant Proteins

Sf9 (Fall Armyworm Ovary; Gibco) cells were cultured and infected with recombinant baculoviruses for expression of Gbeta_1_gamma_2_ as described previously [12]. Recombinant Gbeta_1_gamma_2_ was purified as detailed elsewhere [13]. Purified proteins were quantified by Coomassie Brilliant Blue staining following SDS/PAGE (10% acrylamide) with BSA as the standard. The proteins were stored at −80°C.

### Lipid Kinase Activity Assays

Myc-tagged wild type or E633K p110β was expressed with p85 in HEK 293T cells. The cells were lysed in 120 mM NaCl, 20 mM Tris (pH 7.5), 1 mM MgCl_2_, 1 mM CaCl_2_, 10% glycerol, 1% NP40, containing EDTA-free Protease inhibitor cocktail (Roche) and Phosphatase inhibitor cocktails 1 and 2 (Sigma), and myc-p110β was immunoprecipitated and assayed as described [14]. For assays with purified Gβγ, 200 nM Gβγ was preincubated with lipid vesicles for 30 min and then added to the resuspended enzyme pellets, as described [15]. For assays with phosphopeptide, 1 µM tyrosyl phosphorylated peptide (mouse PDGFR 735–767, sequence ESDGG(pY)MDMSKDESID(pY)VPMLDMKGDIKYADIE; referred to as pY) and lipid vesicles were added directly.

### Sequence Alignment

Sequence alignment of human p110α and human p110β was done using the T-Coffee alignment software (www.tcoffee.org).

### Western Blotting

NIH 3T3 cells stably expressing wild type or E633K p110β were cultured in 6-well dishes for 24 hours then switched to the specified media for an additional 24 hours. Cells were then washed once in PBS and lysed directly in SDS sample buffer. Whole cell lysates were then analyzed by western blotting and blots were visualized using ECL (GE).

### MTT Proliferation Assays

The MTT assay (Invitrogen) was performed as described by the manufacturer. Briefly, 1×10^3^ cells were plated in 96-well plates in the appropriate media. At various times, the cells were incubated with a 12 mM MTT solution in PBS for 4 h at 37°C. An equal volume of 0.1 g/ml SDS solution in 0.01 M HCl was added, and absorbance was read at 570 nm using a Spectramax M5 plate reader (Molecular Devices). For experiments with TGX-221, the cells were treated with 200 nM of TGX-221 throughout the duration of the experiment.

### Trypan Blue Dye Exclusion

Cells were cultured in 6-well dishes (1×10^5^ cells/well) in DMEM/10% NCS for one day and then maintained for 24 hours in DMEM with the specific amount of NCS. Cells were then trypsinized and mixed at 1∶1 volume with 0.4% Trypan Blue Dye. Trypan Blue positive (dead) cells were expressed as a percentage of the total number of cells.

#### Transformation assays

Assays were performed as described in [7]. Briefly, stably-transfected NIH 3T3 cells expressing WT or E633K p110β were plated (2,500 cells/well) in 1 ml of 0.3% top agar over 1 ml of 0.6% bottom agar, in a six-well dish. Cell colonies were counted 3 weeks later. For experiments with inhibitors, the cells were treated with 200 nM of TGX-221, 200 ng/ml of Pertussis toxin, or 30 µM of peptides throughout the duration of the experiment.

### Focus Formation Assays

Assays were performed as described in [7]. Briefly, stably transfected NIH 3T3 cells expressing WT or E633K p110β were plated (2×10^5^ cells/well) in six-well dishes and grown for two weeks, with media (DMEM/10% NCS) being changed every two days. The cells were stained with crystal violet and transformed foci/well counted.

### Boyden Chamber

Stably-transfected NIH 3T3 cells expressing WT or E633K p110β were starved overnight and then plated at 5×10^4^ cells either in serum free or 10% NCS medium in the upper chamber of tissue culture inserts containing 8.0 µm pores (Becton Dickinson and Company, NJ), with DMEM/10% NCS media in the lower chamber. After 5 hours, the cells were fixed in 4% paraformaldehyde. The insert membranes were removed, stained and mounted on coverslips using Dapi Fluoromount (Southern Biotech, AL). Images were collected at 10x magnification using a Nikon Diaphot inverted fluorescence microscope and a SPOT Idea digital camera, and analyzed using ImageJ software. For experiments with TGX-221, the cells were treated with 200 nM of TGX-221 throughout the duration of the experiment.

### Statistical Analysis

Statistical significance was determined using student’s t-test (at http://faculty.vassar.edu/lowry/VassarStats.html).

## Results

### E633K Mutation Increases Basal p110β Activity and Signaling

**Figure 1:**
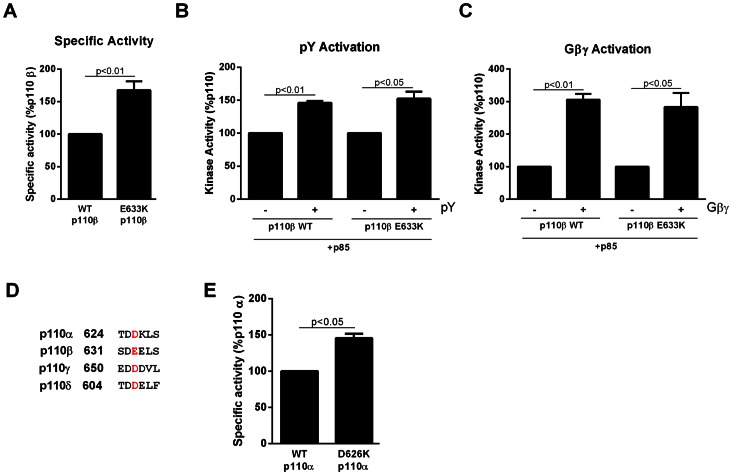
Characterization of the lipid kinase activity of the p110β mutant. (A) HEK 293T cells were transfected with p85 and wild type or E633K myc-p110β. Anti-myc immunoprecipitates were analyzed by western blotting and for lipid kinase activity. (B) Anti-myc immunoprecipitates from cells transfected as above were incubated for 2 hours with pY-peptide and assayed for lipid kinase activity. (C) Anti-myc immunoprecipitates from cells transfected as above were incubated with lipid vesicles/Gβ_1_γ_2_ subunits for 10 minutes and assayed for lipid kinase activity. (D) Sequence alignment of p110α, p110β, p110γ and p110δ focusing on the acidic patch containing the E633 p110β residue, highlighted in red. (E) Specific activity of wild-type and D626K p110α co-expressed with p85 in HEK 293T cells and assayed as above. All data are mean ± SEM of triplicate determination from three separate experiments.

A tumor-associated p110β mutation was identified in a human HER2-positive breast tumor [11]. This mutation, E633K, was not homologous to any previously identified p110α mutation or other mutations identified in the same study in p110γ and p110δ [11]. We generated the mutant p110β and compared its activity to that of wild-type p110β. In an *in vitro* lipid kinase assay, E633K p110β mutant showed a 70% increase in basal activity compared to wild-type p110β (Figure 1A). Both wild type and E633K mutant p110β were activated to a similar extent by a bisphosphotyrosine peptide (pY) (Figure 1B) and Gβγ subunits (Figure 1C).

Using multiple sequence alignment between the four class I catalytic subunits, we observed that the E633 residue in p110β lies in an acidic patch that is conserved in all four class I isoforms (Figure 1D). To test whether mutating this residue in another isoform would have a similar effect on kinase activity, we generated a D626K mutant of p110α. Similar to the p110β E633K mutation, the D626K mutant of p110α showed increased basal kinase activity by ∼50%, compared to wild-type p110α (Figure 1E).

### Mutant p110β Enhances Proliferation, Survival in Low Serum, Transformation Potential and Motility

**Figure 2:**
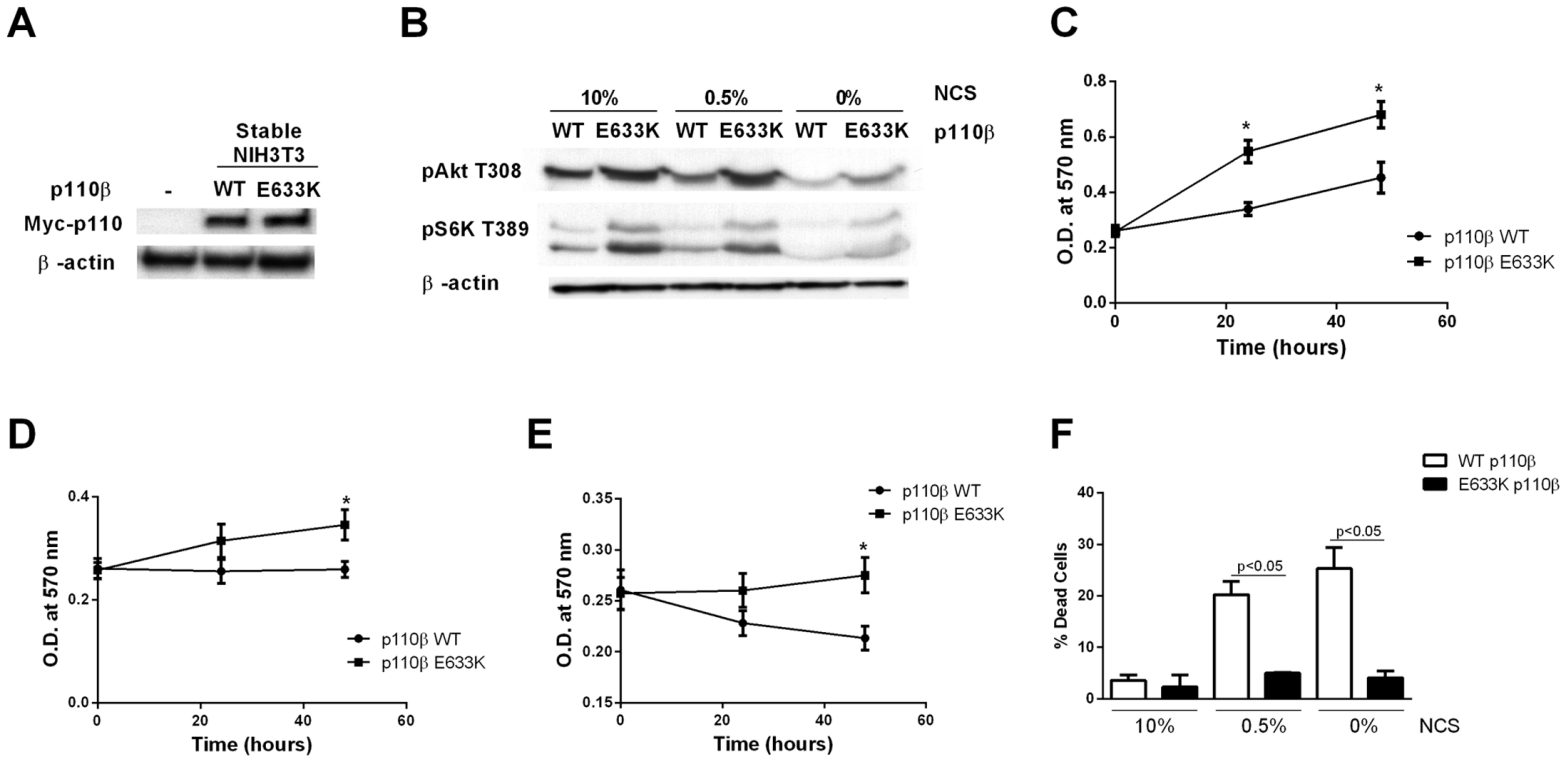
Akt signaling, proliferation and survival of cells expressing mutant p110β. (A) Expression level of wild-type or E633K myc-p110β in stably-transfected cells. (B) Cells stably expressing wild type or E633K p10β were incubated overnight in 10%, 0.5% or 0% NCS media. Whole cell lysates were analyzed by western blotting with anti-pT308 Akt, anti-pT389 S6K, and anti-β-actin antibodies. (C-E) Cells stably expressing wild-type or E633K p110β were plated in 96-well plates, incubated for 24 and 48 hours in (C) 10% NCS medium, (D) 0.5% NCS medium, or (E) 0% NCS medium, and assayed using the MTT assay. (F) Cells stably expressing wild type or E633K p110β were incubated for 24 hours in 10%, 0.5%, or 0% NCS medium. Cell viability was assayed by Trypan blue staining. Dead cells are displayed as percent of total number of cells. Data are mean ± SEM of triplicate samples from two separate experiments.

We generated NIH3T3 cells that stably over-express wild type or E633K mutant p110β (Figure 2A). Cells expressing E633K p110β showed higher levels of basal pT308-Akt and pT389-S6K in 10% NCS and also under low (0.5% NCS) or serum-starved (0% NCS) conditions (Figure 2B). These data show that this mutation enhances the basal activity of p110β *in vitro* and *in vivo*.

Cells expressing E633K p110β showed significantly increased proliferation as compared to cells expressing wild-type p110β under normal growth conditions of 10% serum (Figure 2C). Similarly, in 0.5% serum and 0% serum conditions, cells expressing E633K p110β showed increased proliferation as compared to cells expressing wild-type p110β, which decreased in number over time (Figure 2D, E). Cell death in cells expressing E633K-p110β was decreased as compared to wild type p110β, as detected by a Trypan Blue dye exclusion assay (Figure 2F).

**Figure 3:**
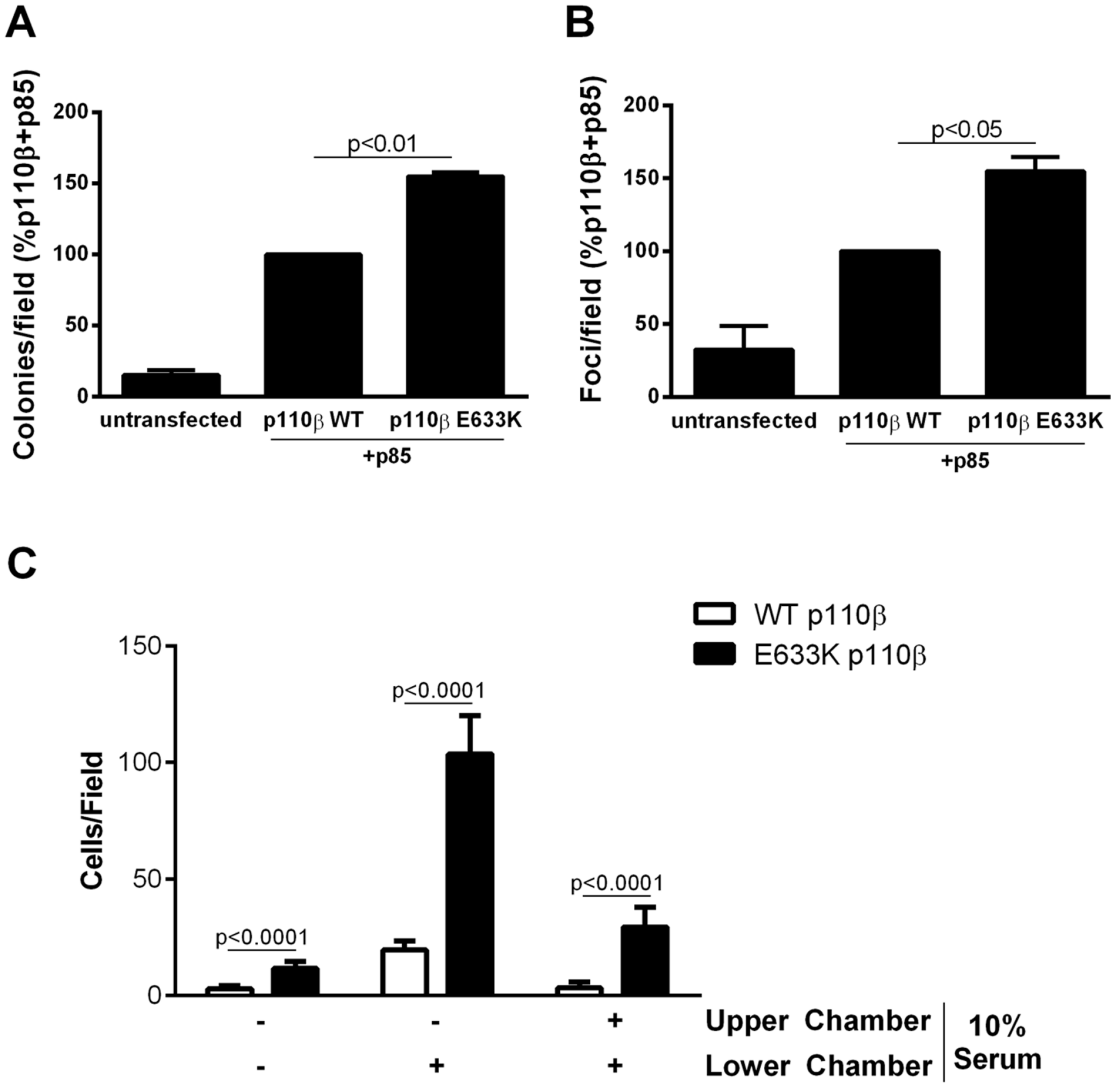
Effect of p110β mutant on transformation and chemotaxis. (A) NIH 3T3 cells stably expressing wild type or E633K p110β were plated in soft agar and colonies were counted after 3 weeks. Colony counts are normalized to the number of colonies produced by cells expressing p110β alone. (B) Equal number of NIH 3T3 cells stably expressing wild type or E633K p110β were plated and left to grow to confluence for 10 days. Foci were counted and normalized to cells expressing wild-type p110β. (C) NIH 3T3 cells stably expressing wild type or E633K p110β were starved overnight and plated either in 0% or 10% NCS in transwell chambers, and incubated with media containing 0% or 10% NCS in the lower chamber and upper chambers as indicated. Data are mean ± SEM of triplicate samples from two experiments.

**Figure 4:**
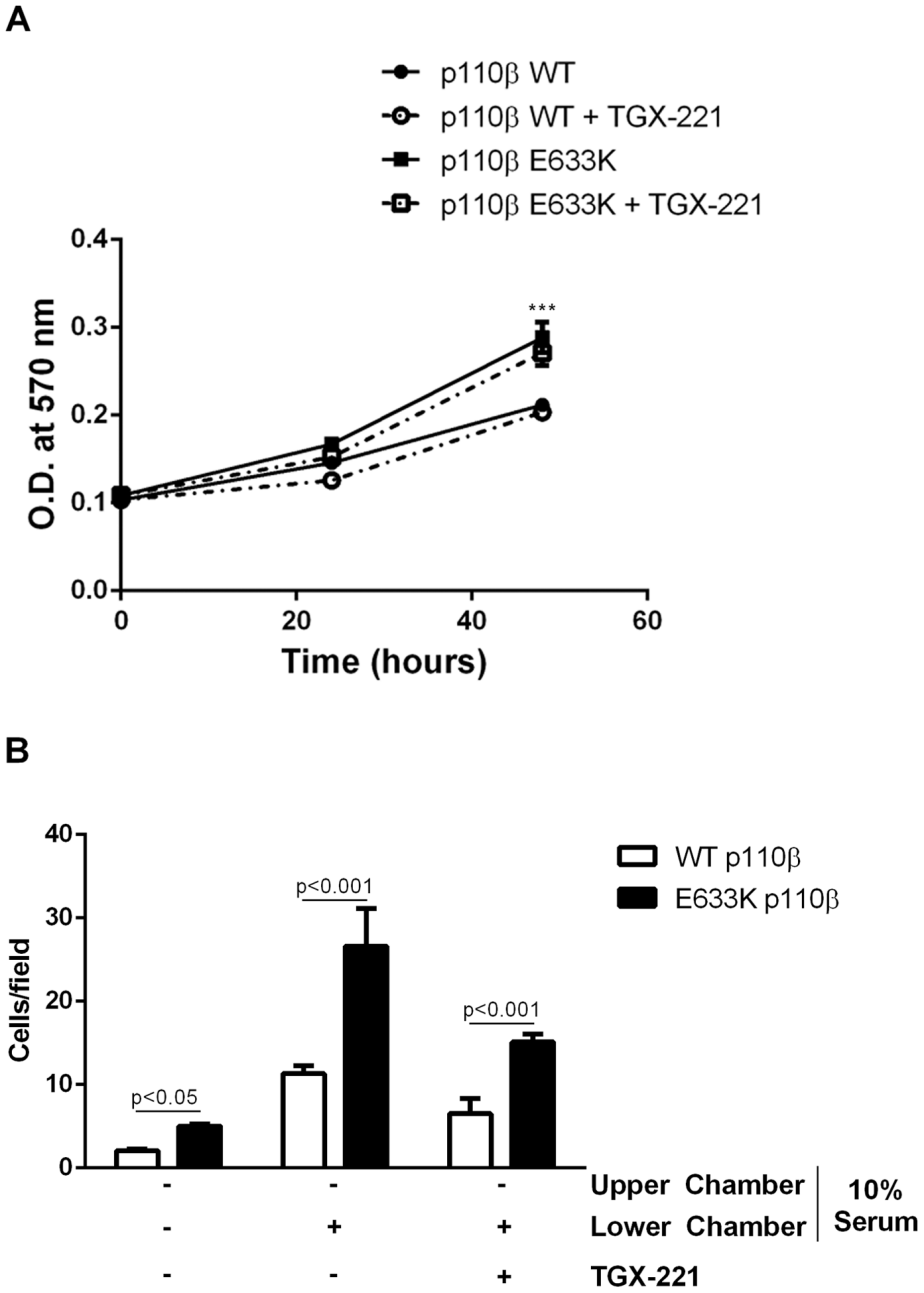
Role of kinase activity on the increased proliferation and migration by the p110β mutant. (A) Cells stably expressing wild-type or E633K p110β were plated in 96-well plates, incubated for 24 and 48 hours in 10% NCS, with or without 200 nM TGX-221, and assayed using the MTT assay. (B) NIH 3T3 cells stably expressing wild type or E633K p110β were starved overnight and plated either in 0% or 10% NCS in transwell chambers, and incubated with media containing 0% or 10% NCS in the lower chamber and upper chambers as indicated, with or without 200 nM TGX-221 in both chambers as indicated. Data are mean ± SEM of at least duplicate samples from two separate experiments.

Over-expression of wild-type p110β is transforming [6]. We tested the effect of the E633K mutation on the transforming potential of p110β *in vitro*. Cells expressing E633K-p110β showed enhanced colony formation in a soft-agar assay as compared to cells expressing wild-type p110β (Figure 3A). Similar results were obtained in a focus formation assay, where cells expressing E633K p110β produced a larger number of foci than cells expressing wild-type p110β (Figure 3B). The increased activity of cells expressing E633K p110β in transformation assays may be due in part their enhanced proliferation rate. Cells expressing E633K mutant p110β also showed increased motility compared to cells expressing wild-type p110β in the absence of serum, in the presence of a serum gradient, or in the presence of serum in both chambers (Figure 3C). Interestingly, the increased proliferation of cells expressing the E633K p110β mutant was unaffected by treatment of cells with TGX-221 (Figure 4A). TGX-221 reduced the migration of cells expressing both wild type and mutant p110β, but the cells expressing mutant p110β still showed a greater than 2-fold enhancement of chemotaxis toward serum (Fig. 4B). These findings are consistent with previous data showing that p110β-dependent proliferation in PC3 cells was independent of kinase activity [10], and suggest that the roles of p110β in proliferation and chemotaxis are due in part to scaffolding functions.

### Transformation by E633K p110β is Unaffected by Inhibition of Ras or Gβγ Binding

**Figure 5:**
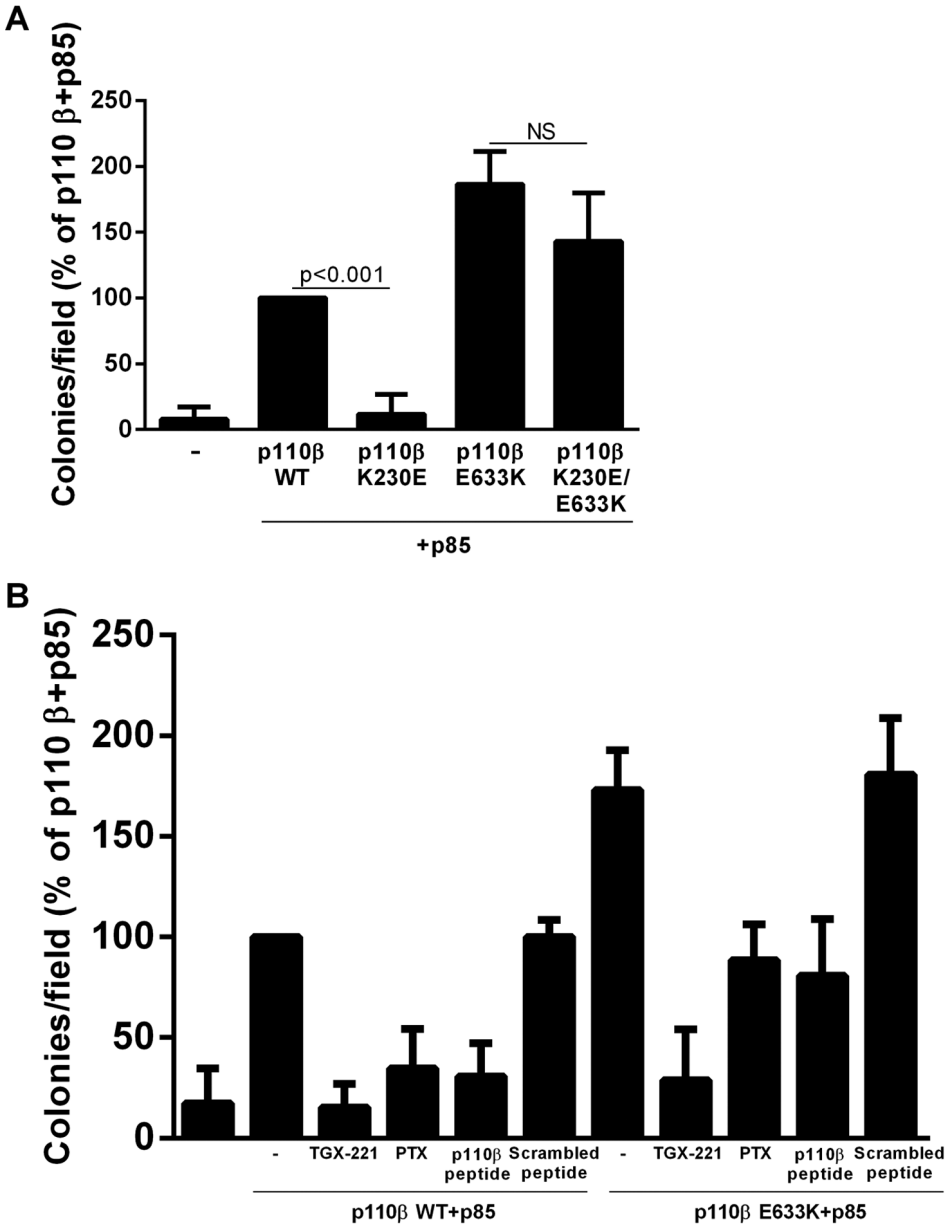
Role of RBD and Gβγ binding on transformation by the p110β mutant. (A) NIH 3T3 cells expressing wild type, K230E, E633K, or K230E/E633K p110β were plated in soft agar and colonies were counted after 3 weeks. Colony counts are normalized to the number of colonies produced by cells expressing p110β alone. (B) NIH 3T3 cells stably expressing wild-type or E633K p110β were plated in soft agar, in regular media or media containing 200 nM TGX-221, 200 ng/ml PTX, or 30×µM of inhibitory peptide or its scrambled counterpart, and colonies were counted after 3 weeks. Colony counts are normalized to the number of colonies produced by cells expressing p110β alone. Data are mean ± SEM of triplicate samples from two separate experiments.

In order to probe the mechanism behind the enhanced transformation of the E633K p110β mutant, we generated a second mutation in the RBD, K230E. The RBD is thought to regulate Class IA PI 3-kinases at least in part by targeting them to the membrane via binding to membrane-associated Ras [16]. Consistent with this, transformation by the H1047R mutant of p110α, which increases membrane binding, is unaffected by a mutation that disrupts Ras binding, whereas transformation by the E545K mutant of p110α requires an intact RBD [17], [18]. Interestingly, the K230E RBD mutation inhibits transformation driven by wild type p110β ([6]) but has no significant effect on transformation in the E633K p110β (Figure 5A). This is consistent with a conformational change leading to enhanced membrane targeting of p110β.

We further tested the requirement for Gβγ binding in the transforming potential of the E633K p110β mutant. We find that the p110β-specific kinase inhibitor, TGX-221, completely abolished transformation by both wild type and E633K p110β (Figure 5B). Transformation by wild-type p110β was also blocked by pertussis toxin (PTX) or by a p110β-derived membrane permeant peptide that blocks p110β binding to Gβγ [10]. In contrast, PTX and the peptide decreased but did not abolish the transformation driven by E633K mutant p110β (Figure 5B). Since binding to Gβγ enhances the association of p110β with membranes [10], the decreased dependence of E633K p110β-mediated transformation on Gβγ is consistent with an enhancement of membrane binding by the mutation.

## Discussion

This study provides the first analysis of a tumor-associated mutation of p110β. The mutation, E633K in the helical domain of p110β, increases basal activity and signaling to Akt and S6K. Expression of the E633K p110β mutant enhances proliferation, survival in low nutrient conditions, transformation and motility, as compared to expression of wild type p110β.

While we have not directly demonstrated an increase in membrane binding for E633K p110β, our experiments are consistent with this hypothesis. First, we find that unlike wild type p110β, transformation by E633K p110β is unaffected by a second mutation in the RBD. Furthermore, unlike wild type p110β, transformation by E633K p110β is only partially inhibited by pertussis toxin or by a cell permeant peptide that inhibits p110β binding to Gβγ. Both Ras and Gβγ subunits are lipidated and reside in the plasma membrane, as well as other intracellular membranes, and a significant component of their activation of PI 3-kinases involved membrane targeting [19]. The ability of E633K p110β to transform cells in the absence of RBD-mediated or Gβγ-mediated inputs strongly suggests that the mutation leads to enhanced membrane targeting. This is analogous to the H1047R mutant of p110α, which shows a decreased dependency on Ras due to its enhanced binding to cell membranes [6], [20], [21].

Unlike transformation in cells expressing wild type or mutant p110β, which is blocked by TGX221, the effects of the E633K p110β mutation on proliferation and motility are to a large part independent of p110β catalytic activity. This is similar to our previous finding that that proliferation of PC3 cells was blocked by inhibition of p110β-Gβγ interactions, but not by treatment with TGX221. In both cases, the effects of enhanced p110β membrane association, due to mutation or Gβγ binding, appear to be at least in part independent of kinase function, suggesting a scaffolding function that is regulated by membrane targeting [10].

E633 is in an acidic patch in the helical domain of p110β, but it juxtaposes the C-terminal end of the ABD-RBD linker. A change in the conformation of this region is characteristic of p85/p110 activation, and the N-terminal end of the ABD-RBD linker shows an increase in membrane association in activated p110α [22]. Given the apparent effects of the E633K mutant on p110β membrane interactions, it is possible that the E633K mutant causes a conformational change in the ABD-RBD linker that increases membrane binding in the mutant p110β. Alternatively, given its proximity to the RBD, it might also act by altering the orientation of this domain within p110β.

E633 is conserved among all class I catalytic subunits, and mutations at the homologous site in p110α also lead to increased activity. It will be interesting to see if mutations of the homologous residues in p110α, p110δ, or p110γ are detected in cancers. The study that identified the E633K p110β mutation also found mutations in p110δ (V397A) and p110γ (N66K, D161E, R178L, S348I, K364N, T503M, R542W, E602V, and E740K) [11]. Interestingly, none of these mutations coincide with regions commonly mutated in p110α, suggesting possible different mechanisms of activation. It will be interesting to study these mutations and assess their effects on kinase activity and transformation by these isoforms, as they may shed new light on the regulation of these isoforms.
